# Attenuative Effects of Platelet-Rich Plasma on 30 kDa Fibronectin Fragment-Induced MMP-13 Expression Associated with TLR2 Signaling in Osteoarthritic Chondrocytes and Synovial Fibroblasts

**DOI:** 10.3390/jcm10194496

**Published:** 2021-09-29

**Authors:** Hsien-Tsung Lu, Jeng-Wei Lu, Chian-Her Lee, Yi-Jen Peng, Herng-Sheng Lee, You-Hsiang Chu, Yi-Jung Ho, Feng-Cheng Liu, Pei-Hung Shen, Chih-Chien Wang

**Affiliations:** 1 Department of Orthopedics, School of Medicine, College of Medicine, Taipei Medical University Hospital, Taipei Medical University, Taipei 110, Taiwan; lu7788@tmu.edu.tw (H.-T.L.); chianherlee@yahoo.com.tw (C.-H.L.); 2 Antimicrobial Resistance Interdisciplinary Research Group, Singapore-MIT-Alliance for Research and Technology, Singapore 138602, Singapore; jengweilu@gmail.com; 3 Department of Pathology, Tri-Service General Hospital, National Defense Medical Center, Taipei 114, Taiwan; yijen0426@gmail.com; 4 Department of Pathology and Laboratory Medicine, Kaohsiung Veterans General Hospital, Kaohsiung 813, Taiwan; herngsheng131419@gmail.com; 5 Graduate Institute of Life Sciences, National Defense Medical Center, Taipei 114, Taiwan; joseph12825@gmail.com (Y.-H.C.); ejung330@gmail.com (Y.-J.H.); 6 School of Pharmacy, National Defense Medical Center, Taipei 114, Taiwan; 7 Rheumatology/Immunology and Allergy, Department of Medicine, Tri-Service General Hospital, National Defense Medical Center, Taipei 114, Taiwan; lfc10399@yahoo.com.tw; 8 Department of Orthopedics, Tri-Service General Hospital, National Defense Medical Center, Taipei 114, Taiwan; sph1468@yahoo.com.tw

**Keywords:** platelet-rich plasma, fibronectin fragment, Toll-like receptor, matrix metalloproteinase, chondrocyte, meniscal fibrochondrocytes, synovial fibroblasts

## Abstract

Proteolytic fragments of fibronectin can have catabolic effects on cartilage, menisci, and synovium. Previous studies have reported that Toll-like receptor (TLR) signaling pathways might be associated with joint inflammation and joint destruction. Platelet-rich plasma (PRP) is increasingly being used to treat a range of joint conditions; however, it has yet to be determined whether PRP influences fibronectin fragment (FN-f) procatabolic activity and TLRs. In this study, human primary culture cells were treated with 30 kDa FN-f with/without PRP co-incubation, and then analyzed using real-time PCR to determine gene expression levels in articular chondrocytes, meniscal fibrochondrocytes, and synovial fibroblasts. Protein levels were evaluated by Western immunoblotting. This study observed an increase in the protein expression of matrix metalloproteinases (MMPs), Toll-like receptor 2 (TLR2), nitric oxide synthase 2 (NOS2), prostaglandin-endoperoxide synthase (PTGS2), and cyclooxygenase 2 (COX2) in articular chondrocytes, meniscal fibrochondrocytes, and synovial fibroblasts following insult with 30 kDa FN-f. Upregulation of these genes was significantly attenuated by PRP treatment. TLR2 and matrix metalloproteinase 13 (MMP-13) were also significantly attenuated by cotreatment with 30 kDa FN-f + PRP + TLR2 inhibitor. PRP treatment was shown to attenuate the 30 kDa FN-f-induced MMP-13 expression associated with the decreased expression of TLR2 in osteoarthritic chondrocytes and synovial fibroblasts. PRP treatment was also shown to attenuate procatabolic activity associated with MMP-13 expression via the TLR2 signaling pathway.

## 1. Introduction

Osteoarthritis (OA) is the most common form of arthritis among the elderly. The onset of cartilage degeneration (the hallmark of OA) can be traced to trauma caused by incorrect biomechanical loading of the joint and consequent biological responses. Previous studies have determined that Toll-like receptors (TLRs) play an important role in the activation of innate immunity by recognizing specific patterns of microbial components [1]. Mature chondrocytes express Toll-like receptor 1 (TLR1) and Toll-like receptor 2 (TLR2) and may react to cartilage matrix/chondrocyte-derived danger signals or degradation products. This leads to the synthesis of proinflammatory cytokines, which further stimulate TLR and cytokine expression, thereby establishing a vicious cycle, eventually leading to OA [2]. Previous studies have demonstrated that the expression of TLR2 in human articular cartilage can be upregulated using proarthritic agents, including interleukin 1 beta (IL-1β) and fibronectin fragment (FN-f) [3]. Signaling through TLR in human articular chondrocytes revealed a novel proinflammatory mechanism associated with OA, indicating that the targeting of these signaling pathways might be of value in the treatment of degenerative joint disease.

FN-f is a multidomain glycoprotein present in most extracellular matrices (ECMs), including cartilage [4] and synovium [5] (in the form of dimeric glycoprotein). FN-f has shown potent catabolic activity associated with an increase in the expression of TLRs and inflammatory cytokines, such as tumor necrosis factor alpha (TNF-α), IL-1β, and interleukin 1 alpha (IL-1-α), as well as the elevated production of matrix metalloproteinase (MMP) proteases and suppressed proteoglycan synthesis [3,6,7,8,9]. Platelet-rich plasma (PRP) is a key source of bioactive molecules used in tissue repair and regeneration. PRP contains a number of growth factors that are important to the regulation of chondrocyte proliferation and anabolic function, including platelet-derived growth factor (PDGF), transforming growth factor beta (TGF-β), fibroblast growth factor (FGF), and insulin-like growth factor 1 (IGF-1) [10,11]. Thus, it is reasonable to surmise that delivering a cocktail of agents, such as those in PRP, could be beneficial to the repair of cartilage and meniscal injury through the stimulation of anabolic activity as well as inhibition of proinflammatory and catabolic pathways [12,13,14].

The current lack of therapeutic solutions to OA progression can be attributed to our poor understanding of the molecular pathophysiology of preclinical and clinical symptomatic OA. We hypothesize that innate immunity and the associated receptors (and especially TLRs) are major drivers of the onset and progression of OA disease. It is likely that this process begins as a proinflammatory reaction against ECM-derived damage-associated molecular patterns (DAMPs). Note that the accumulation of DAMPs in avascular articular cartilage as a result of trauma and/or degeneration leads directly to the chondrocyte-mediated and TLR-dependent production of proinflammatory and algogenic secondary mediators, which in turn cause secondary synovitis with consequent joint pain.

The aim of this study was to assess whether PRP has inhibitory effects on the expression of proinflammatory and proteolytic molecules induced by FN-f via the TLR signaling pathway in human articular chondrocytes, meniscal fibrochondrocytes, and synoviocytes.

## 2. Materials and Methods

### 2.1. Materials and Antibodies

30 kDa FN-fs was purchased from Sigma-Aldrich (St. Louis, MO, USA). Matrix metalloproteinase-1 (MMP-1) and matrix metalloproteinase-3 (MMP-3) antibodies were purchased from Abcam (Cambridge, MA, USA). TLR2 was obtained from Cell Signaling Technology (Danvers, MA, USA), and β-actin antibodies were obtained from Santa Cruz Biotechnology (Dallas, TX, USA). Secondary antibodies were obtained from DAKO Envision+ System (Dako, Glostrup, Denmark).

### 2.2. Patients

Human meniscus, cartilage, and synovial tissue samples were discarded specimens from knee joint arthroplasty surgery on patients with OA (*n* = 53, mean age 75.67 years, range 58−84 years; male: *n* = 40, female: *n* = 13). Note that informed consent was obtained for the use of the specimens (TSGH IRB No. 1-105-05-075).

### 2.3. Cell Culture

Residual OA cartilage, inner meniscus tissue, and synovium were removed from each joint and pooled. The tissue samples were cut into small fragments, incubated with antimicrobial solution containing 500 IU/mL penicillin (Gibco, Burlington, ON, Canada), 500 mg/mL streptomycin (Gibco, Burlington, ON, Canada) and 2.5 μg/mL Fungizone (Sigma-Aldrich, St. Louis, MO, USA) for 4 h, and then washed with sterile phosphate-buffered saline (PBS) prior to digestion. Cartilage and meniscus were extracted via sequential enzymatic digestion with collagenase type H (Sigma-Aldrich, St. Louis, MO, USA) at 37 °C in a humidifier under 5% CO_2_ for 16–18 h. Prior to digestion, the cartilage and meniscus samples were incubated with 0.25% trypsin (Gibco, Burlington, ON, Canada) for 30 min. Following filtration using a 40-μm cell strainer (Millipore, Billerica, MA, USA), the samples were centrifuged and washed two times with PBS. Extracted cells were resuspended in 10 mL Dulbecco’s modified eagle’s medium/nutrient mixture F-12 HAM medium (DMEM/F-12) (Gibco, Burlington, ON, Canada) supplemented with 10% FBS (Gibco, Burlington, ON, Canada), 100 IU/mL penicillin, and 100 mg/mL streptomycin. To obtain human synovial fibroblasts, synovial tissues were minced, stirred with collagenase type I (Gibco, Burlington, ON, Canada) in serum-free Nutrient Mixture F-12 HAM medium (Gibco, Burlington, ON, Canada) for 18–24 h, then filtered through a 70-μm nylon mesh (Millipore, Billerica, MA, USA) and washed extensively. Chondrocytes were maintained in Nutrient Mixture F-12 HAM medium supplemented with 10% FBS (Sigma-Aldrich, St. Louis, MO, USA), 100 IU/mL penicillin, and 100 μg/mL streptomycin. Cells were seeded in complete medium at a density of 5 × 10^5^ cell/mL in 60 mm Petri dishes (TPP, Trasadingen, Switzerland) and cultured in a humidified incubator under 5% CO_2_ at 37 °C until use in experiments. Synovial fibroblasts between passages 3 and 5 were used.

### 2.4. Human Chondrocyte Cell-Line Culture

Human TC28a2 chondrocyte cells (SCC042, Billerica, MA, USA) were cultured in DMEM (Gibco, Burlington, ON, Canada) supplemented with 10% FBS, 100 IU/mL penicillin, and 100 μg/mL streptomycin. Cells were seeded at 5 × 10^5^ cells/dish and grown as a monolayer in 60 mm tissue culture Petri dishes. The cells were washed with sterile PBS twice, placed in serum-free media for 2 h, co-incubated with 10 times concentration (10×) TIRAP (TLR2 and TLR4) inhibitor peptide set (NBP2-26245, Novus Biologicals, Littleton, CO, USA) and PRP for 2 h, and then co-incubated with 30 kDa fibronectin proteolytic fragments at a concentration of 0.25 mg/mL for 4 h. The set contained TIRAP inhibitor peptide (1 mg of DRQIKIWFQNRRMKWKKLQLRDAAPGGAIVS) and control peptide (1 mg of DRQIKIWFQNRRMKWKK), two peptides for research. After four hours for all treatment groups, the control group showed without treatment; the FF group showed with 30 kDa FN-f treatment; 10CF showed 10× control peptide was used with 30 kDa FN-f treatment; 10IF showed 10× inhibitor peptide was used with 30 kDa FN-f treatment; 10CPF showed 10× control peptide, PRP and 30 kDa FN-f cotreatment; 10IPF showed 10× inhibitor peptide, PRP and 30 kDa FN-f cotreatment.

### 2.5. Experimental Protocol

Human articular chondrocytes, meniscal fibrochondrocytes, and synovial fibroblasts were seeded at 5 × 10^5^ cells/dish and grown as a monolayer for 5 days in 60 mm tissue culture Petri dishes. Cells were washed with sterile PBS twice, placed in serum-free medium for 2 h, and then co-incubated with 30 kDa fibronectin proteolytic fragments to stimulate articular chondrocytes, meniscal fibrochondrocytes, and synovial fibroblasts in vitro at a concentration of 0.5 μg/mL for 24 h. Freeze-dried PRP powder was prepared by Regen Lab and Regenkit (Regen Lab, Lausanne, Switzerland) via centrifugation of peripheral blood, using a thixotropic gel for cell separation and citrate as an anticoagulant.

### 2.6. Extraction of RNA and Real-Time Polymerase Chain Reaction (qPCR)

Total RNA was extracted from cell cultures using TRIzol^®^ RNA Isolation Reagents (Invitrogen, Grand Island, NY, USA). For first-strand cDNA synthesis, 2 μg total RNA was used in a single-round reverse-transcription reaction using a High-Capacity cDNA Reverse Transcription Kit (Applied Biosystems, Foster City, CA, USA). Complement DNA was amplified under the following conditions: 50 °C for 2 min, 95 °C for 10 min, 40 cycles of 95 °C for 15 s, and 60 °C for 60 s, using a ViiA7 real-time PCR system (Applied Biosystems, Foster City, CA, USA). The resulting cycle threshold (Ct) values were normalized to the endogenous control, glyceraldehyde-3-phosphate dehydrogenase (GAPDH), and analyzed using the 2^−∆∆Ct^ method. The Taqman Probes used for the analysis of gene expression are listed in Table 1.

### 2.7. Protein Extraction and Western Blotting

Following stimulation, cells were immediately washed with ice-cold PBS, and proteins were extracted with lysis buffer at 4 °C for 15 min. The lysis buffer contained radioimmunoprecipitation assay buffer (RIPA) (Sigma-Aldrich, St. Louis, MO, USA), 100 μM Na3VO4, and protease inhibitor cocktail table (Roche Diagnostics, Mannheim, Germany). Whole-cell lysates were collected after centrifugation at 15,000 rpm for 10 min. Protein concentrations were determined using the Pierce BCA Protein Assay Kit (Thermo Scientific, Rockford, IL, USA). Equal quantities of protein were loaded onto 10% SDS-polyacrylamide gel for transfer to polyvinylidene fluoride (PVDF) membranes (Millipore, Billerica, MA, USA) after electrophoresis. Membranes were blocked overnight at 4 °C with 2% bovine serum albumin (BSA) (Sigma-Aldrich, St. Louis, MO, USA) in tris-buffered saline with tween-20 (TBST). After washing with TBST, blots were incubated at 4 °C overnight with primary antibodies (TLR2, MMP-1, MMP-3, or MMP-13; dilution: ×1000) diluted in TBST, washed three times with TBST, and then incubated with horseradish peroxidase (HRP)-labeled secondary antibodies (Dilution: ×5000) at room temperature for 1 h. Membranes were rewashed with TBST thoroughly and antibody binding was visualized using the Immobilon™ Western HRP Substrate (Millipore, Billerica, MA, USA) in accordance with the manufacturer’s instructions. Immunoblots were scanned using a UVP BioSpectrum AC image system (UVP, Upland, CA, USA) and quantified using VisionWork LS software (UVP, Upland, CA, USA) to determine the ratio of the target protein to the level with β-actin (Dilution: ×5000), which served as an internal control.

### 2.8. Statistical Analysis

The values are expressed as fold of band intensity of the target gene or protein compared to the GAPDH or β-actin internal controls, respectively. The results are expressed as the mean ± SD. Data were analyzed using Prism 8.0 (GraphPad Software Inc., San Diego, CA, USA). All statistical analysis was performed using the Student’s *t*-test with a *p*-value < 0.05 considered statistically significant.

## 3. Results

### 3.1. PRP-Attenuated FN-f-Induced TLR Gene Expression in Articular Chondrocytes, Meniscal Fibrochondrocytes, and Synovial Fibroblasts from the Primary Cultures

**Figure 1:**
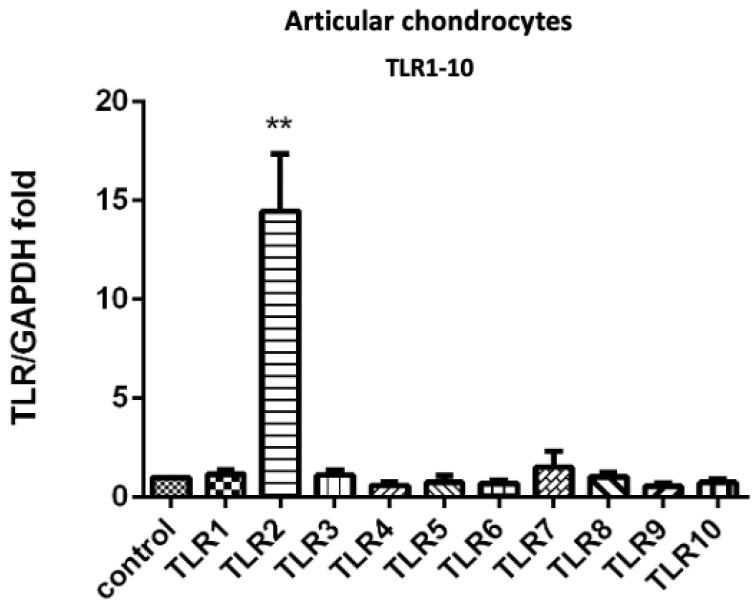
Gene expression of TLR1-10 induced by 30 kDa FN-f in articular chondrocytes. Significant upregulation of the TLR2 gene was observed in human articular chondrocytes (** *p* < 0.001; FN-f treatment compared without FN-f treatment control; *n* = 3, respectively). The other TLR types treated with FN-f showed no significant changes compared to without FN-f treatment control; *n* = 3, respectively. All data are presented as the mean ± SD. Statistical differences among the TLR groups were compared with control values using the Student’s *t*-test.

We first screened the gene expression levels of TLRs on articular chondrocytes after insult with 30 kDa FN-f. We observed a significant increase in TLR2 gene expression, but no other TLR types showed significant differences (Figure 1). To assess the effects of PRP on 30 kDa FN-f-induced TLR1-10 expression in articular chondrocytes, the gene expression of TLRs was determined using qPCR (Figure S1). The observed upregulation of TLR2 in articular chondrocytes by 30 kDa FN-f was suppressed by PRP treatment.

**Figure 2:**
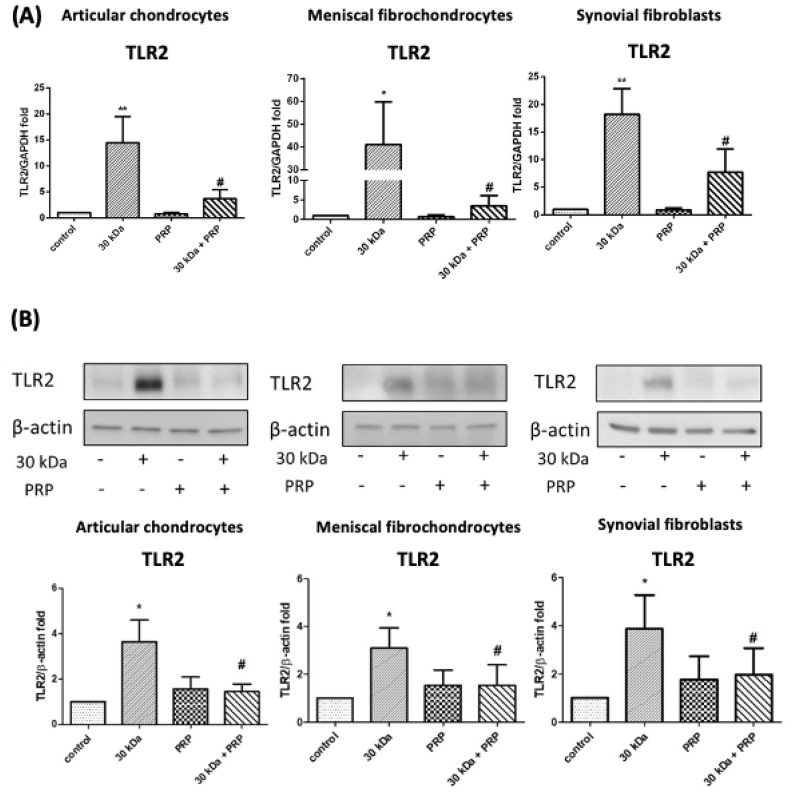
Effects of PRP on 30 kDa FN-f-induced TLR2 gene and protein expression in articular chondrocytes, meniscal fibrochondrocytes, and synovial fibroblasts. (**A**) PRP treatment significantly suppressed TLR2 gene upregulation induced by 30 kDa FN-f in all three type cells. (* *p* < 0.05, compared with control; ** *p* < 0.01, compared with control; # *p* < 0.05, 30 kDa + PRP vs. 30 kDa; *n* = 5 for each group). (**B**) PRP treatment significantly suppressed TLR2 protein upregulation induced by 30 kDa FN-f in all three type cells. Representative blots are presented in the upper panel, and semiquantitative data are presented in the lower panel. All data are presented as the mean ± SD. Statistical differences among TLR groups were compared with control values using the Student’s *t*-test (* *p* < 0.05, compared with control; # *p* < 0.05, 30 kDa + PRP vs. 30 kDa; *n* = 5 for each group).

Experiments based on qPCR were performed to determine whether similar effects occurred in meniscal fibrochondrocytes and synovial fibroblasts. Following incubation with 30 kDa FN-f for 24 h, we observed a significant increase in TLR2 gene expression in chondrocytes, meniscal fibrochondrocytes, and synovial fibroblasts (*p* = 0.009, *p* = 0.024, and *p* = 0.003, respectively). The changes were attenuated by cotreatment with PRP (*p* = 0.02, *p* = 0.024, and *p* = 0.043, respectively) (Figure 2A). We also examined protein expression levels after incubation with 30 kDa FN-f for 24 h. A significant increase in TLR2 protein expression was observed in chondrocytes, meniscal fibrochondrocytes, and synovial fibroblasts (*p* = 0.041, *p* = 0.015, and *p* = 0.014, respectively). These changes were also attenuated by cotreatment with PRP (*p* = 0.02, *p* = 0.042, and *p* = 0.044, respectively) (Figure 2B).

### 3.2. PRP-Attenuated FN-f-Induced MMPs Expression in Articular Chondrocytes, Meniscal Fibrochondrocytes, and Synovial Fibroblasts

**Figure 3:**
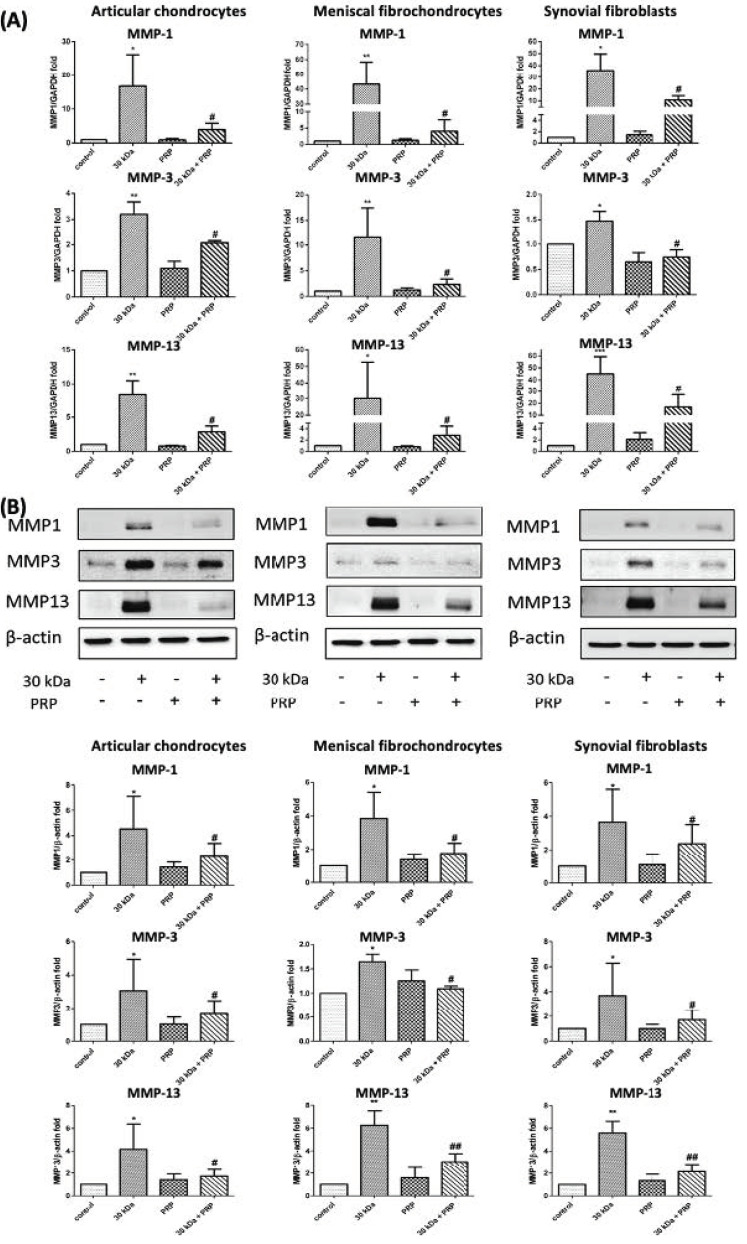
Effects of PRP on 30 kDa FN-f-induced MMP gene and protein expression in articular chondrocytes, meniscal fibrochondrocytes, and synovial fibroblasts. (**A**) Significant suppression of MMP-1, MMP-3, and MMP-13 was observed in all three cell types. (* *p* < 0.05, compared with control; ** *p* < 0.01, compared with control; *** *p* < 0.001, compared with control; # *p* < 0.05, 30 kDa + PRP vs. 30 kDa; *n* = 5 for each group). (**B**) Similar responses were observed in protein levels. Representative blots are presented in the upper panel, and semiquantitative data are presented in the lower panel. All data are presented as the mean ± SD. Statistical differences among the TLR groups were compared with control values using the Student’s *t*-test (* *p* < 0.05, compared with control; ** *p* < 0.01, compared with control; # *p* < 0.05, 30 kDa + PRP vs. 30 kDa: ## *p* < 0.01, 30 kDa + PRP vs. 30 kDa; *n* = 5 for each group).

In a previous study, we demonstrated that MMP gene expression could be increased in both meniscal fibrochondrocytes and articular chondrocytes by treatment with 30 kDa FN-f compared to a control [15]. In the current study, we rechecked the gene expression of MMP-1, MMP-3, and MMP-13 for use as a positive control. We found that 30 kDa FN-f insult significantly increased MMP-1 gene expression in articular chondrocytes, meniscal fibrochondrocytes, and synovial fibroblasts (*p* = 0.04, *p* = 0.0048, and *p* = 0.048, respectively). Similar results were obtained for MMP-3 (*p* = 0.0028, *p* = 0.044, and *p* = 0.0046, respectively) and MMP-13 (*p* = 0.0054, *p* = 0.006, and *p* = 0.049, respectively) (Figure 3A).

In subsequent experiments, cotreatment with PRP was shown to attenuate the increased protein expression of MMP-1 (*p* = 0.037, *p* = 0.0119, and *p* = 0.038, respectively), MMP-3 (*p* = 0.015, *p* = 0.026, and *p* = 0.049, respectively), and MMP-13 (*p* = 0.014, *p* = 0.08, and *p* = 0.006, respectively) in articular chondrocytes, meniscal fibrochondrocytes, and synovial fibroblasts (Figure 3A). In experiments on MMP protein expression, we also observed significant increases in MMP-1, MMP-3, and MMP-13 in articular chondrocytes, meniscal fibrochondrocytes, and synovial fibroblasts (*p* = 0.0305, *p* = 0.035, and *p* = 0.0206 for MMP-1, respectively) (*p* = 0.0073, *p* = 0.02, and *p* = 0.015 for MMP-3, respectively) (*p* = 0.0254, *p* = 0.0035, and *p* = 0.0029 for MMP-13, respectively) (Figure 3B).

In experiments involving cotreatment with PRP, we again observed the attenuation of changes in protein expression in articular chondrocytes, meniscal fibrochondrocytes, and synovial fibroblasts, as follows: MMP-1 (*p* = 0.0350, *p* = 0.046, and *p* = 0.027, respectively), MMP-3 (*p* = 0.043, *p* = 0.029, and *p* = 0.013, respectively), MMP-13 (*p* = 0.044, *p* = 0.0043, and *p* = 0.0013, respectively) (Figure 3B).

### 3.3. Expression of TLR2 and MMP-13 under FN-f Insult in Chondrocyte Cell Line

**Figure 4:**
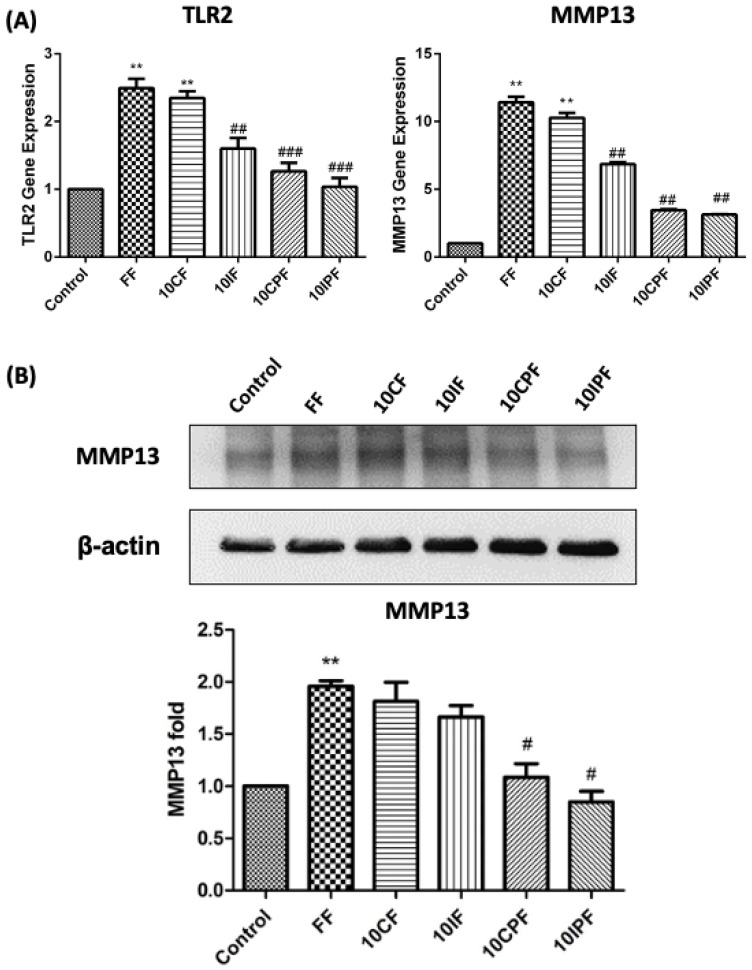
Effects of PRP on MMP-13 regulation associated with TLR2 pathway in chondrocyte cell line. We used 10× TLR2 peptide antagonist to test (**A**) TLR2 and MMP-13 gene regulation in chondrocyte cells. FF treatment and 10× CF groups were significantly higher compared with control groups. The 10× CFP and 10× IPF groups were significantly lower compared with FF groups. (**B**) Representative Western blotting indicates the effects of FF incubated with inhibitor peptide and PRP. Cotreatment with 10IPF resulted in MMP-13 expression far below that of the FF groups. All data are presented as the mean ± SD. The Student’s *t*-test was used to compare statistical differences among the following groups: FF groups and 10CF groups were compared with control values; 10IF groups, 10CPF groups, and 10IPF groups were compared with FF groups (FF: 30 KDa fibronectin fragment, CF: Control peptide; IF: Inhibitor peptide, CPF: Control peptide + PRP + FF; IPF: Inhibitor peptide + PRP + FF); *n* = 5 for each group; ** *p* < 0.05, # *p* < 0.05, ## *p* < 0.01, ### *p* < 0.001.

The inhibitor of TLR2 peptide was used for the effects of FN-f on TLR2 and MMP-13 gene or protein expression levels. After incubating chondrocyte cells with 30 kDa FN-f (FF) for 24 h, we observed a significant increase in the gene expression of TLR2 (*p* = 0.0086). Incubation with the 10× control peptide (10CF) for 24 h prompted a significant increase in TLR2 gene expression in chondrocyte cells (*p* = 0.004). Incubation with the 10× inhibitor peptide (10IF) for 24 h resulted in TLR2 gene expression significantly below that of the 30 kDa FN-f group (*p* = 0.0005). Cotreatment using PRP + control peptide (10CPF) for 24 h also resulted in TLR2 gene expression significantly lower than that of the 30 kDa FN-f group (*p* = 0.0007). Finally, cotreatment with PRP + inhibitor peptide (10IPF) for 24 h also resulted in TLR2 gene expression significantly lower than that of the 30 kDa FN-f group (*p* = 0.003) (Figure 4A).

We also used qPCR to examine the expression of the MMP-13 gene with 30 kDa FN-f treatment in the chondrocyte cell line. Incubation with 30 kDa FN-f (FF) for 24 h prompted a significant increase in MMP-13 gene expression (*p* = 0.0015). Treatment with 10× control peptide (10CF) for 24 h significantly increased MMP-13 gene expression (*p* = 0.0074). Treatment with 10× inhibitor peptide (10IF) resulted in MMP-13 gene expression significantly lower than that of the 30 kDa FN-f group (6.85 ± 0.28-fold, *p* = 0.0043). Cotreatment with PRP + control peptide (10CPF) resulted in MMP-13 gene expression significantly lower than that of the 30 kDa FN-f group (*p* = 0.003). Finally, cotreatment with PRP + inhibitor peptide (10IPF) also resulted in MMP-13 gene expression significantly lower than that of the 30 kDa FN-f group (*p* = 0.0018) (Figure 4A).

### 3.4. FN-f-Induced MMP-13 Protein Expression in Chondrocyte Cells Was Attenuated by Cotreatment with PRP

We also characterized the protein expression of MMP-13 in chondrocyte cells to determine whether PRP attenuates 30 kDa FN-f-induced inflammation through the activation of MMP-13. As shown in Figure 4B, treating chondrocyte cells with 30 kDa FN-f (FF) for 24 h resulted in MMP-13 protein expression significantly exceeding that of the control group (*p* = 0.0032). Treatment with 10× control peptide (10CF) or 10× inhibitor peptide (10IF) did not have a significant effect on MMP-13 protein expression levels. Cotreatment with PRP + control peptide (10CPF) showed significant attenuation of MMP-13 protein expression compared with 30 kDa FN-f (FF) group (*p* = 0.03). Cotreatment with PRP + inhibitor peptide (10IPF) resulted in MMP-13 protein expression significantly lower than that of the 30 kDa FN-f (FF)group (*p* = 0.043) (Figure 4B).

### 3.5. PRP Attenuates FN-f-Induced Catabolism Gene Expression in Meniscal Fibrochondrocytes, Articular Chondrocytes, and Synovial Fibroblasts

**Figure 5:**
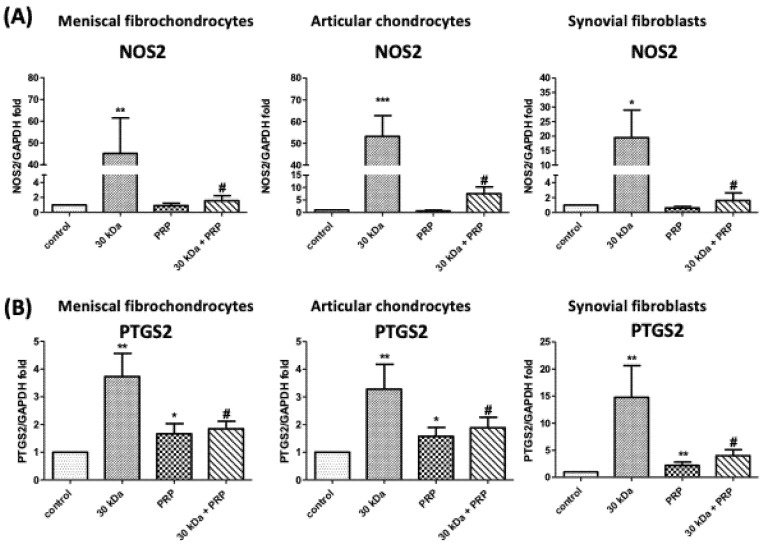
Effects of PRP on 30 kDa FN-f-induced NOS2 and PTGS2 gene expression in meniscal fibrochondrocytes, articular chondrocytes, and synovial fibroblasts. (**A**) PRP treatment suppressed upregulation of NOS2 and (**B**) PTGS2 (* *p* < 0.05, compared with control; ** *p* < 0.01, compared with control; *** *p* < 0.001, compared with control; # *p* < 0.05, 30 kDa + PRP vs. 30 kDa; *n* = 5 for each group).

Incubation with 30 kDa FN-f for 24 h significantly increased the gene expression of nitric oxide synthase 2 (NOS2) in meniscal fibrochondrocytes, chondrocytes, and synovial fibroblasts (*p* = 0.009, *p* = 0.0007, and *p* = 0.0284, respectively) and cyclooxygenase 2 (COX2) (*p* = 0.0048, *p* = 0.0022, and *p* = 0.0033, respectively). Cotreatment with PRP attenuated the effects on COX2 (*p* = 0.02, *p* = 0.02, and *p* = 0.011, respectively) and NOS2 (*p* = 0.0095, *p* = 0.0013, and *p* = 0.032, respectively) (Figure 5).

### 3.6. FN-f-Induced NOS2 and COX-2 Protein Levels by Meniscal fibrochondrocytes, Articular Chondrocytes, and Synovial Fibroblasts Are Inhibited by PRP

**Figure 6:**
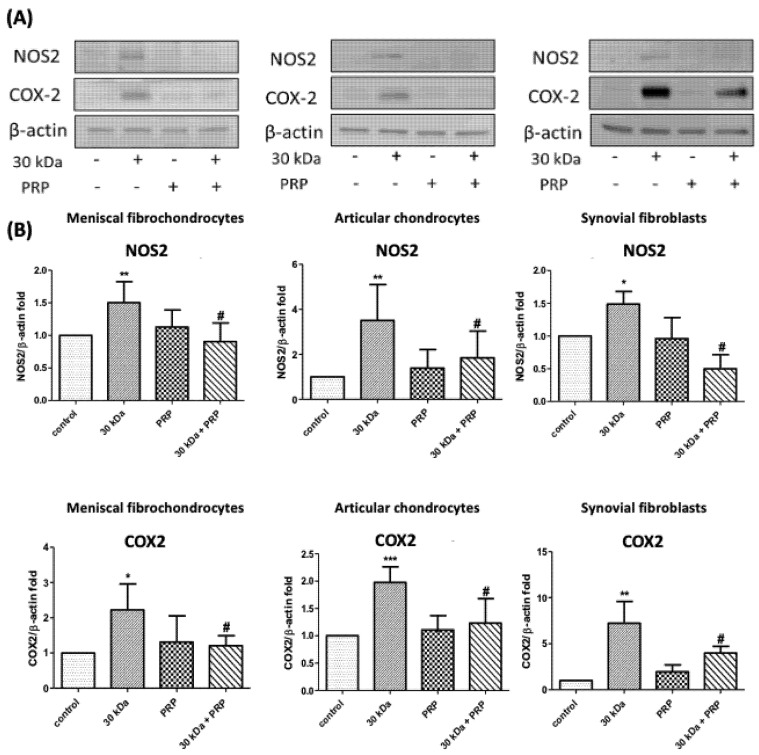
Effects of PRP on 30 kDa FN-f-induced COX2 and NOS2 protein expression in meniscal fibrochondrocytes, articular chondrocytes, and synovial fibroblasts. (**A**,**B**) PRP treatment suppressed upregulation of COX2 and NOS2 induced by 30 kDa FN-f (* *p* < 0.05, compared with control; ** *p* < 0.01, compared with control; *** *p* < 0.005, compared with control; # *p* < 0.05, 30 kDa + PRP vs. 30 kDa; *n* = 5 for each group).

Incubation with 30 kDa FN-f for 24 h significantly increased NOS2 (*p* = 0.008, *p* = 0.0061, and *p* = 0.042, respectively) and COX-2 (*p* = 0.044, *p* = 0.0016, and *p* = 0.013, respectively) protein levels in meniscal fibrochondrocytes, chondrocytes, and synovial fibroblasts. Cotreatment with PRP attenuated these effects on NOS2 (*p* = 0.0143, *p* = 0.047, and *p* = 0.031) and COX-2 (*p* = 0.041, *p* = 0.014, and *p* = 0.04, respectively) (Figure 6).

## 4. Discussion

In previous research, we characterized the effects of 30 kDa FN-f in stimulating the expression of proinflammatory chemokines and MMPs, including interleukin 8 (IL-8), interleukin 6 (IL-6), C-C motif chemokine ligand 20 (CCL20), C-C motif chemokine ligand 5 (CCL5), C-X-C motif chemokine ligand 10 (CXCL10), MMP-1, MMP-3, and MMP-13 in human articular chondrocytes and meniscal fibrochondrocytes [15]. We also demonstrated that these effects could be attenuated by PRP treatment. Here, we assessed the influence of PRP on FN-f-induced proinflammation states in three major cells of the knee joint (articular chondrocytes, meniscal fibrochondrocytes, and synovial fibroblasts). FN-f was shown to increase the expression of MMPs and TLR2 in all three cell types. Co-stimulation with PRP was shown to significantly attenuate the FN-f-induced catabolic effects on MMP and TLR2 expression. FN-f binding to integrin stimulates chondrocyte-mediated cartilage destruction and thus may play an important role in the progression of arthritis [16]. The ability of FN-f to stimulate the expression of multiple proinflammatory cytokines and chemokines suggests that damage to the cartilage matrix induces a proinflammatory state leading to further progressive destruction of the matrix [16]. FN-fs generated by proteolytic cleavage of FN are found at elevated levels in OA cartilage and synovial fluid [17].

A number of endogenous matrix molecules, including fibronectin (FN), have recently been found to mediate cartilage degradation [7]. FN-fs have been studied extensively due largely to their affinity for cartilage matrix molecules [18]. Each FN-f possesses specific proteolytic activities; however, FN-f-induced cartilage catabolism is due primarily to an increase in the expression levels and activity of MMP [8]. In in vitro studies, FN-f has been shown to cause cartilage damage by promoting the release of proteases [18,19]. FN-f has been identified as the most potent inducer of cartilage catabolism, where the injection of 30 kDa FN-f into rabbit knee joints resulted in a loss of up to 70% of the cartilage proteoglycan, in vivo [6]. It has been shown that 29 kDa FN-f can induce MMPs as well as procatabolic cytokines, including IL-1α, IL-1β, IL-6, and TNF-α, in addition to nitric oxide (NO) in human and bovine cartilage [20]. Targeting the signaling pathways activated by FN-f may be an effective approach to inhibiting the production of multiple mediators of cartilage destruction [16]. In our study, FN-f was shown to increase TLR2 expression levels in articular chondrocytes (Figure 1 and Figure 2), indicating that FN-f may play an important role in articular chondrocyte inflammation associated with the progression of OA.

The mammalian TLR family includes 13 members, which recognize specific arrangements of microbial components, referred to as pathogen-associated molecular patterns (PAMPs). TLR-dependent recognition of PAMPs leads to activation of the innate immune system, which subsequently leads to activation of antigen-specific adaptive immunity [21]. TLRs have been implicated in a variety of cellular responses, such as defensive actions [22]. TLR signaling is crucial to the functional activation of immune responses during infection [23]. Overall, the TLR family is best characterized as a group of innate immune receptors in terms of known ligands, functional relevance, and downstream signaling pathways [24]. TLR signaling has been implicated in the pathogenesis of sepsis, asthma, atherosclerosis, and autoimmune disorders [25,26,27,28]. TLR signaling pathways can be traced back to intracytoplasmic TIR domains (conserved among all TLRs) and MyD88, which is essential to the induction of inflammatory cytokines [1]. TLRs recognize PAMPs, including bacterial peptidoglycan (PGN) and lipopolysaccharide (LPS), and damage-associated molecular patterns (DAMPs), which are released upon tissue injury [1].

Thus, the inhibition of TLR signaling pathways may have beneficial effects in terms of joint inflammation and joint destruction [29]. It appears likely that TLR2 expression in chondrocytes is upregulated by a noninfectious, inflammatory pathway, thereby raising the possibility that this family of molecules contributes to the detrimental inflammatory/catabolic activities of chondrocytes in OA [3,30]. This study identified signaling via TLRs as a novel proinflammatory mechanism underlying OA. Thus, it appears that targeting these signaling pathways may provide benefits in treating degenerative joint disease [3]. Numerous studies have compiled evidence suggesting that the cellular TLR levels and/or the expression of TLRs may be related to the homeostasis of cartilage matrix. Researchers have previously reported on elevated TLR levels in articular cartilage and cultured chondrocytes in cases of OA [3,29].

TLR2 and TLR4 ligands can induce strong catabolic responses in chondrocytes [31]. Specifically, TLR2 expression is upregulated by catabolic cytokines, including IL-1β and TLR2 ligands [3,31], and TLR2 can recognize endogenous ligands [32]. Note that hyaluronan fragments increase proinflammatory cytokine levels by activating CD44 and TLR4 in chondrocytes. Thus, it appears that DAMP recognition by TLR may play a role in progressive cartilage damage [33]. In our present study of meniscal fibrochondrocytes in addition to chondrocytes and synovial fibroblasts, expression of TLR2 on exposure to the proinflammatory 30 kDa FN-f is a novel observation. For the treatment effect of PRP, 30 kDa FN-f induced-TLR2 protein levels by meniscal fibrochondrocytes, articular chondrocytes, and synovial fibroblasts could be attenuated. Our use of chondrocyte cells in experiments on TLR2 and MMP-13 gene expression was meant to prevent the death of primary human articular chondrocytes after treatment with TLR2 inhibitor peptide. Our finding that 30 kDa FN-f significantly upregulated TLR2 gene expression in OA cartilage and synovial fibroblasts suggests a strong relationship between catabolic signaling induced by FN-fs and TLR2 (Figure 4).

MMPs are a family of zinc- and calcium-dependent endopeptidases with broad proteolytic capabilities, involved in remodeling the extracellular matrix of connective tissue [34,35]. The activity of endogenous MMPs is normally inhibited by endogenous tissue inhibitors of metalloproteinases (TIMPs) [34]. MMP inhibitors have been shown to reduce the severity of cartilage degradation in OA [36,37]. Cartilage breakdown results in the production of metabolically active breakdown products, such as FN-f. Thus, it appears likely that FN-f can also be produced in menisci following acute or chronic degeneration, with corresponding effects on all intra-articular tissues. The 45 kDa Fn-f was able to modulate the gene expression of MMP-13 by porcine articular chondrocytes, and also stimulated the synthesis of MMP-3 from cultured chondrocytes and cartilage cultures [8]. In OA-fibroblast-like synoviocytes, 45 kDa Fn-f mediated production of MMP-9 and MMP-13 by enzyme-linked immunosorbent assay (ELISA) [38]. Our previous study demonstrated that the mRNA expression of MMP-1, MMP-3, and MMP-13 genes could be increased in both meniscal fibrochondrocytes and articular chondrocytes by treatment with 30 kDa FN-f, compared to a control [15]. Here, our observation of MMP-1, MMP-3, and MMP-13 gene expression in meniscal fibrochondrocytes as well as chondrocytes and synovial fibroblasts upon exposure to proinflammatory 30 kDa FN-f, is a novel finding. These results suggest that PRP treatment could reduce FN-f-induced MMP-1, MMP-3, and MMP-13 protein-expression levels in meniscal fibrochondrocytes, articular chondrocytes, and synovial fibroblasts. Western blotting analysis revealed that cotreatment with 30 kDa FN-f + PRP + TLR2 inhibitor had a more pronounced effect than did 30 kDa FN-f + TLR2 inhibitor in attenuating MMP-13 gene expression levels (Figure 3). This means that PRP treatment may attenuate 30 kDa FN-f-induced MMP-13 gene expression via the TLR2 signaling pathway.

Preclinical studies have shown that PRP enhances chondrocyte viability, proliferation, and matrix production [39,40]. PRP has recently been posited as a treatment for OA; however, researchers have yet to determine the relative importance or optimal concentration of each PRP component. At present, it appears that platelet concentrations roughly 2.5 to 3 times higher than baseline levels are ideal, as higher concentrations could potentially inhibit tissue healing [41,42]. In treating OA of the knee, PRP is generally injected at concentrations of 2 to 6 times higher than normal platelet concentrations; however, researchers have yet to determine the optimal protocol [43]. The platelet concentration of the commercial PRP preparation used in the current study was 3.3 times higher than the normal concentration (i.e., within the ‘therapeutic’ range). Based on the data obtained in the current study, it appears that PRP attenuated the effects of FN-f on the production of MMPs in chondrocytes, meniscal fibrochondrocytes, and synovial fibroblasts. The mechanism underlying these effects remains unclear; however, it is likely multifactorial (i.e., affecting numerous overlapping pathways), due to the presence of multiple anti-inflammatory cytokines and anabolic growth factors [12]. Chondrocytes are the single-cellular component of hyaline cartilage. Under physiologic conditions, chondrocytes remain in a steady-state equilibrium between anabolic and catabolic activities to maintain the structural and functional integrity of the cartilage extracellular matrix. Implicit in the loss of cartilage matrix associated with OA is a disturbance in the regulation of synthetic (anabolic) and resorptive (catabolic) activities of resident chondrocytes, resulting in a net loss of cartilage matrix components and deterioration of the structural and functional properties of the cartilage [44].

The IL-1 stimulates the production of proinflammatory mediators involved in articular inflammation, such as prostaglandin E2 (PGE2) and NO, which have been implicated in the pathogenesis of OA, in vitro [45,46]. PGE2 upregulates MMPs that cause joint cartilage degradation [47]. PGE2 exerting antianabolic effects has been shown to affect human adult articular cartilage, and EP2 and EP4 receptor antagonists are potential therapeutic agents for the treatment of OA [48]. During inflammation, the overproduction of NO can damage chondrocytes. NO is also considered a catabolic factor responsible for perpetuating OA progression [46]. NO has been shown to have an impact on cartilage homeostasis [49] and also induces MMP synthesis in articular chondrocytes [50]. NO from exogenous and endogenous sources can induce apoptotic insults to chondrocytes via a mitochondria-dependent mechanism [49]. Furthermore, NO can trigger apoptosis, which appears to be mediated through the regulated expression of *caspase 3* and *7* [51,52]. Thus, it appears that antagonists of NO production promote synthesis of the cartilage matrix and could therefore be used as chondroprotective or chondroreparative agents.

This was the first study to observe the 30 kDa FN-f-induced gene expression of prostaglandin-endoperoxide synthase (PTGS2) and NOS2 in meniscal fibrochondrocytes as well as chondrocytes and synovial fibroblasts. Nonetheless, the effects of this increase in the expression of cytokines in cartilage menisci and synovial fibroblasts have yet to be elucidated. The results showed that FN-f-induced COX-2 and NOS2 protein levels by meniscal fibrochondrocytes, articular chondrocytes, and synovial fibroblasts is inhibited by PRP treatment (Figure 5 and Figure 6). In the current study, we demonstrated that PRP attenuated the effects of such an environment on human osteoarthritic chondrocytes and synovial fibroblasts. Unfortunately, this short-term in vitro model was insufficient to study PRP as a possible treatment for OA. Based on these limitations, in order to explore the potential functions and mechanisms of OA and its relationship with PRP, it is necessary to further analyze the function of PRP in OA based on long-term in vivo experiments.

## 5. Conclusions

In the current study, 30 kDa FN-f was shown to induce the overexpression of TLR2 and catabolic factors, including MMP-1, MMP-3, and MMP-13 in articular chondrocytes, meniscal fibrochondrocytes, and synovial fibroblasts. We also showed that PRP could be used to attenuate these effects. It appears that the above-mentioned catabolic responses can be traced to the TLR-2 signaling pathway in chondrocytes and synovial fibroblasts. Thus, our findings provide further evidence that the TLR2-mediated signaling pathway contributes to cartilage matrix degradation in OA, and thus may serve as a therapeutic target for OA. Our observations indicate that the mechanism of PRP may ameliorate the progression of OA, and that the current use of PRP for joint damage needs to be further determined.

## Figures and Tables

**Table 1:** TaqMan probes for gene expression analysis.

Gene Name	Accession Number	Applied Biosystems Number
TLR1	NM_003263	Hs00413978_m1
TLR2	NM_003264	Hs00610101_m1
TLR3	NM_003265	Hs01551078_m1
TLR4	NM_003266	Hs00152939_m1
TLR5	NM_003268	Hs00152825_m1
TLR6	NM_006068	Hs00271977_s1
TLR7	NM_016562	Hs00152971_m1
TLR8	AF246971.1	Hs00152972_m1
TLR9	NM_017442	Hs00152973_m1
TLR10	NM_001017388	Hs00999403_m1
MMP1	NM_002421	Hs00233958_m1
MMP3	NM_002422	Hs00968305_m1
MMP13	NM_002427	Hs00233992_m1
GAPDH	NM_002046	Hs02758991_g1

## Data Availability

Data is contained within the article or Supplementary Material.

## Supplementary Materials

The following are available online at https://www.mdpi.com/article/10.3390/jcm10194496/s1, Figure S1: Gene expression of TLR1-10 induced in articular chondrocytes via treatment with 30 kDa FN-f and PRP.
