# Supplementary material for: Attenuative Effects of Platelet-Rich Plasma on 30 kDa Fibronectin Fragment-Induced MMP-13 Expression Associated with TLR2 Signaling in Osteoarthritic Chondrocytes and Synovial Fibroblasts
Source: J Clin Med. 2021 Sep 29;10(19):4496. doi: 10.3390/jcm10194496 (PMC8509240; doi:10.3390/jcm10194496)
Supplement: Supplementary file 1 [file jcm-10-04496-s001.zip › jcm-1387937-supplementary.pdf]

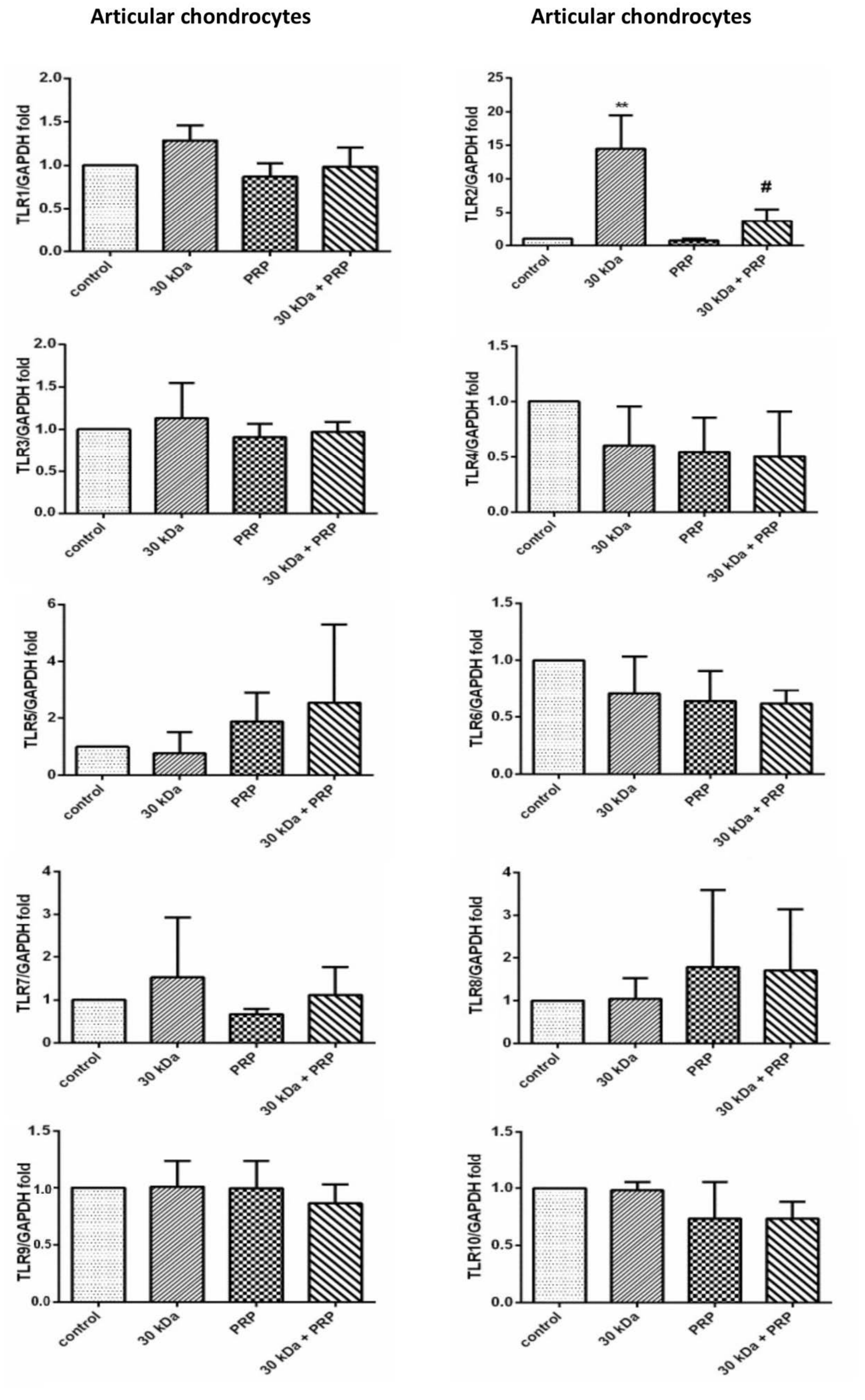

Figure S1. Gene expression of *TLR1-10* induced in articular chondrocytes via

treatment with 30 kDa FN-f and PRP. *TLR* gene expression levels detected using qPCR after incubation with 30 kDa FN-f and PRP for 24 hours. All data are presented as the mean ± SD. Statistical differences among 30 kDa FN-f groups were compared with control values, whereas 30 kDa FN-f+PRP groups were compared with 30 kDa FN-f groups using the student's *t*-test (**$p < 0.01$, compared with control; #$p < 0.05$, 30 kDa+PRP vs 30 kDa; $n = 5$ for each group).
